# Lead exposure dose-dependently affects oxidative stress, AsA-GSH, photosynthesis, and mineral content in pakchoi (*Brassica chinensis* L.)

**DOI:** 10.3389/fpls.2022.1007276

**Published:** 2022-10-06

**Authors:** Zhanming Tan, Cuiyun Wu, Zhengying Xuan, Yunxia Cheng, Renci Xiong, Zhihang Su, Desheng Wang

**Affiliations:** ^1^ College of Horticulture and Forestry Sciences, Tarim University, Alar, China; ^2^ College of Agronomy, Tarim University, Alar, China; ^3^ The National-local Joint Engineering Laboratory for Efficient and High-quality Cultivation and Deep Processing Technology of Characteristic Fruit Tress in Southern Xinjiang, Alar, China

**Keywords:** Pb, pakchoi, dose dependence, AsA-GSH, photosynthesis

## Abstract

Lead (Pb) is a heavy metal pollutant and negatively affects agriculture and ecosystems. Pb can cause oxidative stress and abnormal plant growth. The ascorbic acid-glutathione (AsA-GSH) cycle mainly exists in chloroplasts and resists oxidative stress, scavenges reactive oxygen radicals, and maintains normal photosynthesis. However, the dosage related effects of Pb on pakchoi photosynthesis, *via* oxidative stress and the AsA-GSH system, remains unclear. In this study, various Pb dosage stress models were tested (low: 300 mg/kg; medium: 600 mg/kg; high: 900 mg/kg). Pb stress induced a dose-dependent increase in Pb content in pakchoi leaves (P < 0.05). Principal component analysis showed that Se, B, and Pb were significantly and negatively correlated. Pb stress also increased MDA content and decreased antioxidant enzymes SOD, GSH-Px, and T-AOC activities (P < 0.05). We also found that Vc content, as well as the GSH/GSSG ratio, decreased. Additionally, Pb stress destroyed chloroplast structure, decreased photosynthesis indicators Pn, Tr, Gs, Ci and VPD, and attenuated Fv/Fm and Fv/Fo (P < 0.05). In the high-dose group, the contents of chlorophyll a, chlorophyll b, and carotenoids decreased significantly, while the expression of chloroplast development genes (GLK, GLN2) decreased (P < 0.05). Our data suggest that Pb stress leads to dosage-dependent, aberrant photosynthesis by inhibiting the AsA-GSH system in pakchoi. This study expands the Pb toxicology research field and provides indications for screening antagonists.

## Introduction

Lead (Pb) is a non-essential, heavy metal plant pollutant, derived mainly from waste gas, batteries, and canned products (Ye et al., 2018; Dong et al., 2021). The Pb contents in the Yangtze River and the estuary in winter were 11.3 to 669.4 μg/g in China. From the 1980s to 2016, the Pb content increased by 77-78% due to pollution (Yu et al., 2021). The Pb found in household ash mainly derives from coal burning and solid waste incineration, and is consistent with the Pb levels in urban air and soil surface. Household dust is therefore considered to be the main environmental cause for children requiring treatment for Pb related problems (Dong et al., 2021). Moreover, the Pb content in soils approximately 20 kilometers away from the La Oroya metallurgical complex in Peru was 217.81 ± 39.48 mg/kg, of which 9.5% was transferred to the surrounding grasses (Doris et al., 2021). The Pb content in lettuce leaves and Chinese cabbage grown in urban garden soils reached 0.05 mg/kg fresh weight, and the Pb concentration in these vegetables was positively correlated with the Pb soil levels (Gao et al., 2021; Calabró et al., 2022). The over-standard rate of Pb in 673 plant samples provided by a typical intensive production system in Hainan Province is 2.67%, and leafy vegetables are more polluted than non-leafy vegetables (Yang et al., 2021). Crop plants mainly absorb Pb by absorbing Pb^2+^ ions found in the soil solution. Thus, when the soil becomes acidic, insoluble PbCO_3_ is easily released and absorbed by plants. Most of the Pb absorbed by plants accumulates in the roots, whereafter it then migrates to stems and leaves (Dalyan et al., 2018). Pb can hinder plant root formation, resulting in decreased plant seed germination rates, plant height, leaf number, biomass, and yield (Ye et al., 2018; Kanwal et al., 2020). Thus, excessive soil Pb content threatens plant growth and development, and can even cause plant death. Pb stress is known to reduce chlorophyll pigment and gas exchange characteristics, leading to plant oxidative damage (Bamagoos et al., 2021). After Pb exposure (3000 mg/kg), chlorophyll production, photosynthetic efficiency, and PSII (the reaction center of photosystem II) decreases (Xie et al., 2021). Moreover, Pb exposure affects ascorbic acid metabolism, which results in oxidative and chloroplast damage (Zhang et al., 2020).

In the study of plant stress physiology, the ascorbic acid-glutathione (AsA-GSH) circulatory system participates in resisting oxidative stress and scavenging reactive oxygen free radicals in chloroplasts (Ahmad et al., 2010; Ahmad et al., 2019; Kohli et al., 2019). AsA and reduced GSH levels are important non-enzymatic antioxidants that are closely related to plant stress resistance. In the AsA-GSH cycle, the ratio of AsA/dehydroascorbate (DHA), as well as the ratio of GSH/oxidized glutathione (GSSG), can be used to measure the response of plants to environmental stress (Li et al., 2022). Dehydroascorbate reductase (DHAR), monodehydroascorbate reductase (MDHAR), ascorbate peroxidase (APX), and glutathione reductase (GR), are important enzyme components in the AsA-GSH cycle of plants, and important for regenerating reduced AsA and GSH (Gao and Chen, 2005). Drought stress can affect the antioxidant capacity of cotton leaves. Consequently, AsA and GSH content increases under drought stress, and the ability of the AsA-GSH cycle to eliminate ROS is weakened (Raja et al., 2021). In salt-treated soybeans, malondialdehyde (MDA), hydrogen peroxide (H_2_O_2_), catalase (CAT), MDHAR, and DHAR content increases as salt levels increase (Rahman et al., 2021). Therefore, the AsA-GSH cycle is critical for preventing plant oxidative damage caused by stress.

Pb stress is known to impact plant growth and photosynthesis (Ye et al., 2018; Kanwal et al., 2020; Xie et al., 2021). Pakchoi (*Brassica chinensis* L.) is a type of miniature Chinese cabbage, and is a subspecies of Cruciferae Brassicae. The Chinese populace is fond of pakchoi due to its small size, high nutritional value, and ease of growing. However, whether Pb stress causes a dosage-dependent effect on the mineral element content, AsA-GSH cycle, photosynthesis, and chloroplast development in pakchoi, remains unclear. We therefore studied pakchoi by using low, medium, and high dosage exposure models of Pb stress (Zeng et al., 2007). We also measured whole element content, AsA-GSH cycle levels, antioxidant enzyme activities, photosynthesis, chlorophyll content, and chloroplast development-related genes. This study aimed to elucidate the dosage-dependent influences of Pb stress on the AsA-GSH cycle and photosynthesis in pakchoi to provide a reference for Pb toxicology.

## Methods and materials

### Planting and processing of pakchoi

The pakchoi variety used in this study was “April Slow” (Wanlida, China). The purity of this variety is ≥ 92.0%, and its germination rate is over 98.0%. There were four experimental treatments: control (C), 300 mg/kg Pb (L), 600 mg/kg (M), and 900 mg/kg (H). We used (CH₃COO)_2_Pb from Aladdin (Cat. No. 301-04-2). Three replicates were used for each treatment. In the experiment, 300 mm x 200 mm (upper diameter x height) ceramic pots were used, which were filled with 3.0 kg of soil and Pb mixture. We added deionized water along the pot edges to ensure that the soil moisture content reached the maximum capillary water holding capacity. After standing for 24 h, we planted 15 pakchoi seeds per pot to a depth of 1 cm. Seedlings emerged 3–5 days after sowing, and thinning was performed a week later, thereby leaving six strong seedlings in each pot. We arranged the potted plants randomly and changed their position every day to ensure that each pot received an even amount of light. During the entire experimental period, the temperature was maintained at 18–22°C, the light was kept at 2×10^4^ - 3×10^4^ lux, and the soil moisture content at 50%. The experiment was carried out in the Tarim University greenhouse. The planting date was March 4, 2021, and plants were harvested 45 days later.

The potted soil used is a sandy loam, and its basic characteristics are shown in **Table 1**. A base fertilizer was applied to the soil, and consisted of: 0.33 g/kg urea, 0.10 g/kg potassium dihydrogen phosphate, 0.09 g/kg potassium chloride. No top dressing was applied during the growing period.

**Table 1:** Physical and chemical properties of potting soil.

Soil properties	Potting soil
pH	7.05 ± 0.04
Organic matter (g/kg)	23.5 ± 0.20
Total nitrogen (g/kg)	1.35 ± 0.02
Available phosphorus (mg/kg)	9.05 ± 0.1
Available potassium (mg/kg)	81.51 ± 1.04
Pb (mg/kg)	0.22 ± 0.05

### Inductively coupled plasma mass spectrometry analysis, principal component analysis and correlation analyses

Upon harvesting, cabbage leaves were collected and washed with deionized water, whereafter they were oven dried first at 105°C for 30 min, and then at 60°C. Specific steps are given by Farhat et al. (Farhat et al., 2022). After acid digestion, the following elements were detected using ICP-MS technology (iCAP Q, Thermo): Lithium (Li), Beryllium (Be), Boron (B), Sodium (Na), Magnesium (Mg), Aluminum (Al), Phosphorus (P), Potassium (K), Calcium (Ca), Titanium (Ti), Vanadium (V), Chromium (Cr), Manganese (Mn), Iron (Fe), Cobalt (Co), Nickel (Ni), Copper (Cu), Zinc (Zn), Gallium (Ge), Arsenic (As), Selenium (Se), Rubidium (Rb), Strontium (Sr), Molybdenum (Mo), Silver (Ag), Cadmium (Cd), Tin (Sn), Antimony (Sb), Barium (Ba), Mercury (Hg), Thallium (Tl), Lead (Pb) and Bismuth (Bi).

For the Pb-stressed pakchoi leaves, we used logarithmic (base 10) values for total element content. Hereafter, we used the SPSS (version 25.0) software to perform a PCA through dimensionality reduction. Finally, we used the Origin (version 2021) software for correlation analysis.

### Determination of photosynthetic characteristics

The experiment was carried out between 9:00–11:00 AM on a sunny day with occasional cloud cover. The light intensity was 800 μmol m^−2^ s^−1^, the CO_2_ consistence was 500 μmol mol^−1^, and the humidity was 62%. We randomly selected three healthy pakchoi leaves from each group for photosynthetic index determination and consistently used the same leaf position each time. Assuming that the water pressure difference between the pakchoi leaves and air was 1.0–1.2 kPa, we used a handheld photosynthetic measurement system (LI-6400XT, Lincoln) to gauge the net photosynthetic rate (Pn), transpiration rate (Tr), stomatal conductance (Gs), intercellular CO_2_ concentration (Ci), vapor pressure difference (VPD), and atmospheric CO_2_ concentration (Ca), as well as other pakchoi indicators (Kamran et al., 2019).

### Observation of the ultrastructure of chloroplast

A small piece of 1 mm × 3 mm was cut from the middle of the pakchoi leaf, avoiding the main lateral vein, and immediately placed in 4% glutaraldehyde for pre-fixation, and then post-fixed with 1% glutaric acid. Then, dehydration, infiltration, embedding, aggregation, sectioning and staining (uranyl acetate-lead citrate double staining) were performed according to the conventional ultra-thin sectioning method, and then observed and photographed with a transmission electron microscope (GEM-1200ES, Japan).

### Determination of fluorescence characteristic parameters of pakchoi

According to a previous report (Li et al., 2018), we used a portable chlorophyll fluorometer (FMS-2, UK) to measure the fluorescence parameters of healthy pakchoi leaves under a set light intensity, and using a consistent position for each leaf. Prior to measurement, pakchoi leaves were dark treated for 15 min, and a low light intensity (1 μmol m^-2^ s^-1^) was applied to gauge the initial fluorescence (Fo). Hereafter, we used a saturated pulsed light intensity (3000 μmol m^-2^ s^-1^) to gauge maximum fluorescence (Fm), variable fluorescence (Fv = Fm-Fo), maximum photochemical efficiency of PSII (Fv/Fm), potential of PSII photochemical activity (Fv/Fo), 100 μs photoreaction center closed purification rate (dVG/dto), and 300 μs photoreaction center closed purification rate (dV/dto). Three leaves were chosen for each treatment.

### Determination of photosynthetic pigment content pakchoi leaves

A total of 0.5 g fresh pakchoi leaves were placed in 95% ethanol and protected from light for 24 h to extract photosynthetic pigments. A spectrophotometer (Hitachi UV-3100 UV/VIS; TECHCOMP, China) was used to measure extract absorbances at 665, 649, and 470 nm. Calculation formulas for chlorophyll a, chlorophyll b, and carotenoids were based on a previous report (Mr et al,. 2021). Three leaves were chosen for each treatment.

### AsA and DHA content in pakchoi

We used colorimetric and phenanthroline colorimetric methods to detect the AsA and DHA leaf content in the four treatment groups. Following the manufacturer’s instructions, we used Vitamin C (VC) content (A009-1-1, Jiancheng Nanjing) and DHA (TC2041, Leagene Beijing) test kits.

### Detection of oxidative stress levels in pakchoi

We used a phosphate buffer (pH 7.4) to grind a weighed 0.1 g sample of pakchoi. The supernatant was collected after centrifugation at 3500 r/min for 15 min. Hereafter, we analyzed MDA content, as well as GSH-Px, SOD, T-AOC, GSH, and GSSG activities, according to the manufacturer’s instructions (Nanjing Jiancheng, China). The detailed product numbers were as follows: MDA Determination Kit (Item No. A003-1-2), Glutathione Peroxidase (GSH-Px) Determination Kit (Item No. A005-1-2), Superoxide Dismutase (SOD) Test Kit (Item No. A001-1-2), Total Antioxidant Capacity (T-AOC) Test Kit (Item No. A015-1-2), and Total Glutathione (GSH)/Oxidized Glutathione (GSSG) Determination kit (Item No. A061-2-1).

### Analysis of pakchoi mRNA levels

A TRIzol™ reagent (Item No. 12183555, Invitrogen) was used to collect total RNA from pakchoi leaves based on a previously published method (Xu et al., 2021). We then used a cDNA synthesis kit (BioFlux, China) to reverse transcribe total RNA to cDNA. The primers of the genes detected by quantitative reverse transcription polymerase chain reaction (qRT-PCR) are given in **Table S1**. These primers referenced the genomes of Chinese cabbage (Taxonomy ID: 51351) and Brassica rapa (Taxonomy ID: 3711), which were synthesized by Shanghai Sangon Biotechnology Co., Ltd. An endogenous control, namely β-actin, was used to standardize other target genes. The qRT-PCR reaction program was executed using a SYBR Green fluorescent dye (BioFlux, China) according to the manufacturer’s instructions. Relative mRNA levels and genes were calculated using the 2^−ΔΔCt^ method (Ali Shah et al., 2021).

### Statistical analysis

We used GraphPad Prism (version 8.0) and SPSS (version 25.0) to perform a one-way analysis of variance on all data. The relevant experimental data of the Pb-stressed pakchoi leaves were all normally distributed and passed the equal variance test. The results are expressed in terms of mean ± standard deviation (M ± SD). Groups with the same letter represent non-significant differences; groups with different letters represent significant differences.

## Results

### Pb and total element content of pakchoi leaves

**Figure 1:**
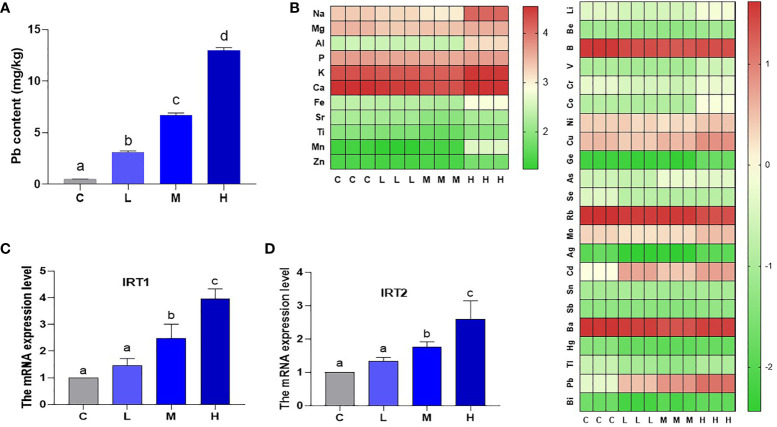
Pb content and total element content of leaves of pakchoi. **(A)** Pb content (mg/kg) in the leaves of pakchoi (45 d). **(B)** After Pb exposure in pakchoi, the heat map of total element content. High expression (red), Low expression (green). **(C)** qRT-PCR analysis of IRT1 mRNA level in pakchoi leaves (45 d) (n=3). **(D)** qRT-PCR analysis of IRT2 mRNA level in pakchoi leaves (45 d) (n=3). The same letter indicates no significant difference (P > 0.05); completely different letter indicates significant difference (P < 0.05).

The Pb content was 0.48518 mg/kg (C group), 3.108726 mg/kg (L group), 6.696257 mg/kg (M group), and 12.96486 mg/kg (H group) as determined by ICP-MS (**Figure 1A**). In the Pb stress group, pakchoi leaf Pb content increased with increasing soil Pb content. IRT (iron-regulated transporter 1), as a member of the Zip family (ZRT IRT-like protein), participates in plant heavy metal ions transport. Here, the levels of IRT1 and IRT2 mRNA in M group increased by 147.3% and 75.0%, respectively, and the levels of IRT1 and IRT2 mRNA in H group increased by 296.3% and 159.1% (P < 0.05), respectively, while the increase in L group was not statistically significant (P > 0.05) (**Figures 1C, D**). These results indicate that soil applied Pb is transported to pakchoi leaves and is dosage-dependent. A heat map of total element content showed that Rb content in the H group decreased, while V, Fe, Cu, Co, Cd, Zn, As, and Mo content increased. It is worth noting that Mn content in the H group increased by nearly twenty-fold (**Figure 1B**).

**Figure 2:**
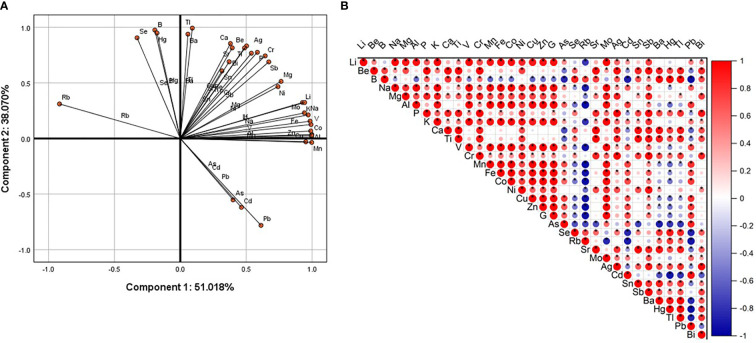
Principal component analysis and correlation analysis. **(A)** Use SPSS (version 25.0) software to carry out the PCA of total element content (logarithm based on 10). The first component (x axis) is 51.018%, and the second component (y axis) is 38.070%. **(B)** Use Origin software to perform correlation analysis on the rotated score matrix output by PCA. Positive correlation (red), negative correlation (blue). The color depth and the size of the circle are related to the strength of the correlation. The “*” sign indicates significant difference.

All-element principal component analysis results showed that Pb, Cd, and As were negatively correlated with Se, Hg, B, and Rb on the first and second components. Pb, Cd, As and Tl, Ba, Ca, Be, Sr, Sn, Bi, Ti, Ag, Cr, P, Sb, Mg, Ni, Mo, Li, K, Na, V, Co, Al were all negatively correlated with component one and positively correlated with component two (**Figure 2A**). Correlation analysis results showed that Pb was strongly and negatively correlated with B, Rb, Se, Ba, Hg, and Tl. Pb had a strong positive correlation with Cd, As, Ge, Zn, Cu, Co, Fe, Mn, V, and Al (**Figure 2B**). These results indicate that increased Pb content reduces the contents of B, Rb, Se, Hg, and Tl, and increases the contents of Cd, As, Ge, Zn, Cu, Co, Fe, Mn, V, and Al.

### The effect of Pb stress on the AsA-GSH cycle of pakchoi

**Figure 3:**
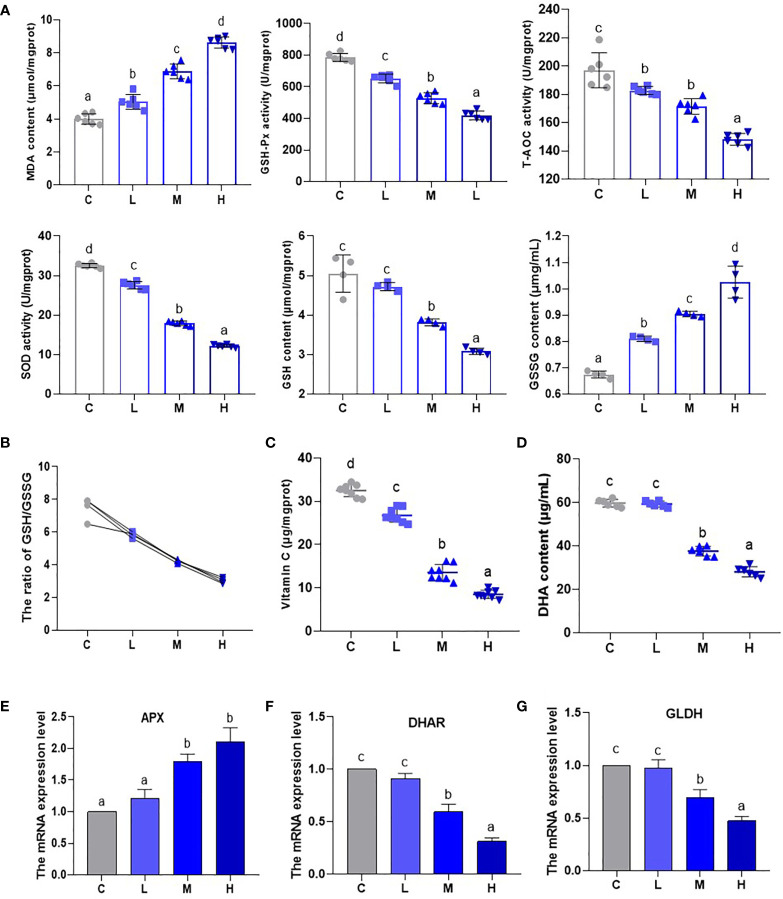
The effect of Pb stress on the AsA-GSH cycle of pakchoi. **(A)** The contents and activities of MDA (μmol/mgprot), GSH-Px (U/mgprot), T-AOC (U/mgprot), SOD (U/mgprot) (n=6), GSH (μmol/mgprot) and GSSG (μmg/mL) (n=4) in pakchoi. **(B)** The ratio of GSH/GSSG (n=4). **(C)** The content of AsA (Vitamin C) (μg/mgprot) in pakchoi leaves (45 d) (n=8). **(D)** The content of DHA (μg/mL) in pakchoi leaves (45 d) (n=8). **(E)** qRT-PCR analysis of APX mRNA level in pakchoi leaves (45 d) (n=3). **(F)** qRT-PCR analysis of DHAR mRNA level in pakchoi leaves (45 d) (n=3). **(G)** qRT-PCR analysis of GLDH mRNA level in pakchoi leaves (45 d) (n=3). The same letter indicates no significant difference (P > 0.05); completely different letter indicates significant difference (P < 0.05).

An analysis of oxidative stress-related indicator (GSH-Px, T-AOC, SOD, GSH, MDA, and GSSG) content and activity (**Figure 3A**) showed that MDA content increased (P < 0.05), while the activities of T-AOC, GSH-Px, and SOD experienced a dosage dependent reduction (P < 0.05). This shows that the oxidative stress levels of pakchoi gradually increase as Pb dosage increases. Additionally, the GSH contents of the M and H groups decreased, while the GSSG content increased significantly to 134.05% and 151.95% of the control group, which appeared to be dosage dependent (P < 0.05) (**Figure 3A**). Moreover, GSH/GSSG, as a measure of plant response to ecological environmental stress, showed a dosage-dependent decrease (P < 0.05) (**Figure 3B**). Subsequently, the contents of AsA and DHA decreased significantly in the M and H groups (**Figures 3C, D**). Glutamate dehydrogenase (GLDH) is a key rate-limiting enzyme for AsA synthesis. APX and DHAR are genes related to the AsA-GSH system. Thus, we analyzed their mRNA levels by qRT-PCR (**Figures 3E–G**). In the M and H groups, the transcription level of APX was up-regulated (P < 0.05) as the Pb dosage increased, while the transcription levels of DHAR and GLDH were down-regulated. These results confirm that the pakchoi AsA-DHA system experiences a state of disorder under Pb stress.

### Pb stress influences pakchoi photosynthesis and is dose-dependent

**Figure 4:**
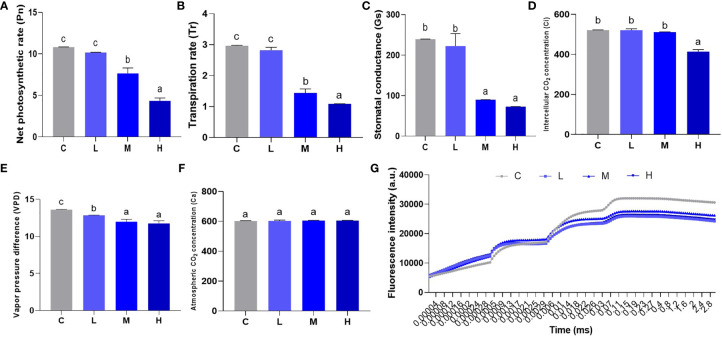
Effect of Pb stress on the dose-dependent effect of photosynthesis of pakchoi. **(A)** The net photosynthetic rate (Pn) in pakchoi leaves (44 d) (n=3). **(B)** The transpiration rate (Tr) in pakchoi leaves (44 d) (n=3). **(C)** The stomatal conductance (Gs) in pakchoi leaves (44 d) (n=3). **(D)** The intercellular CO2 concentration (Ci) in pakchoi leaves (44 d) (n=3). **(E)** The vapor pressure difference (VPD) in pakchoi leaves (44 d) (n=3). **(F)** The atmospheric CO_2_ concentration (Ca) in pakchoi leaves (44 d) (n=3). **(G)** Over time, the fluorescence intensity (a. u.) of pakchoi in pakchoi leaves (44 d) (n=3). The same letter indicates no significant difference (P > 0.05); completely different letter indicates significant difference (P < 0.05).

Photosynthesis uses inorganic matter to produce organic matter and store energy, which forms the basis for plant survival. We used a hand-held photosynthesis meter to detect Pn, Tr, Gs, Ci, VPD, and Ca to study the effect of Pb stress on pakchoi photosynthesis. In the low-dosage Pb treatment, Pn, Tr, and Gs levels did not increase significantly. The values of Pn (**Figure 4A**), Tr (**Figure 4B**), and Gs (**Figure 4C**) reduced significantly (P < 0.05) in the M and H groups, while Gs levels remained similar (P > 0.05). Furthermore, the Ci values of the L and M groups did not change significantly (**Figure 4D**), while H group Ci levels decreased significantly (P < 0.05). Pb treatment slightly decreased the value of VPD (**Figure 4E**), but the dosage dependence was not significant between M and L groups. The atmospheric CO_2_ concentration did not change significantly among the four treatments (**Figure 4F**). Finally, we found that Pb treatment decreased the fluorescence intensity of pakchoi leaves over time (**Figure 4G**). Although no dosage-dependent effect was found, the fluorescence intensity of the low-dose Pb treatment decreased most significantly over time. These results indicate that Pb stress weakens pakchoi photosynthesis, which appeared to be dosage dependent.

### Pb stress has a dose-dependent effect on the PSII response system of pakchoi

**Figure 5:**
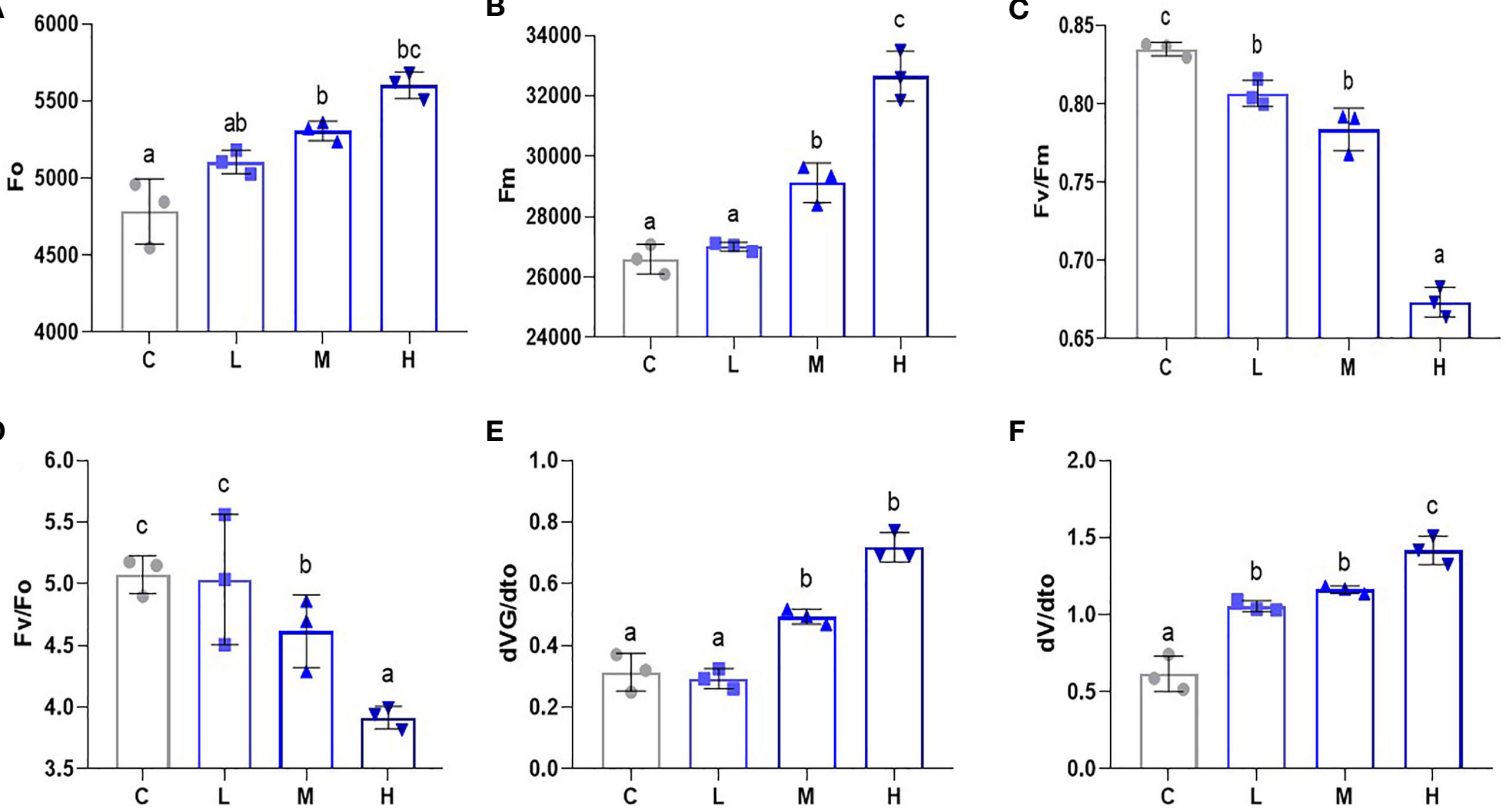
Pb stress has a dose-dependent effect on the PSII response system of pakchoi. **(A)** The initial fluorescence (Fo) of Pb stress pakchoi (44 d) (n=3). **(B)** The maximum fluorescence (Fm) of Pb stress pakchoi (44 d) (n=3). **(C)** The maximum photochemical efficiency of PSII (Fv/Fm) in pakchoi leaves (44 d) (n=3). **(D)** The potential of PSII Photochemical activity (Fv/Fo) in pakchoi leaves (44 d) (n=3). **(E)** The 100 μs photoreaction center closed purification rate (dVG/dto) in pakchoi leaves (44 d) (n=3). **(F)** The 300 μs photoreaction center closed purification rate (dV/dto) in pakchoi leaves (44 d) (n=3). The same letter indicates no significant difference (P > 0.05); completely different letter indicates significant difference (P < 0.05).

To gauge the influence of Pb stress on the PSII reaction system in pakchoi, we used a portable chlorophyll fluorometer to determine the Fo, Fm, Fv/Fm, Fv/Fo, dVG/dto, and dV/dto values. Fo levels increased after Pb treatment (**Figure 5A**). Additionally, we found that Fm increased with an increased Pb dosage (**Figure 5B**), while the medium dosage treatment also showed significant differences (P < 0.05). PSII reaction is the photosynthetic unit in the light-reactive thylakoid membrane. Fv/Fm can be used to measure the original light energy conversion efficiency of PSII. Fv/Fm decreased significantly with increased Pb dosage (P < 0.05) (**Figure 5C**), which indicates that Pb stress inhibited the PSII response system of pakchoi. Additionally, compared with the other three treatments, high-dosage Pb exposure significantly decreased the Fv/Fo value (**Figure 5D**), which means that the maximum light energy conversion potential of PSII reaction was reduced. We then selected the purification rate of extensive initiation center closure at two time points, namely 100 μs and 300 μs (**Figures 5E, F**). Both dVG/dto and dV/dto increased as the Pb dosage increased; however, the dosage dependencies were not obvious. These results indicate that Pb stress has a dosage-dependent inhibitory effect on the PSII response system of pakchoi.

### The influence of Pb treatment on the chlorophyll content and chloroplast formation of pakchoi

**Figure 6:**
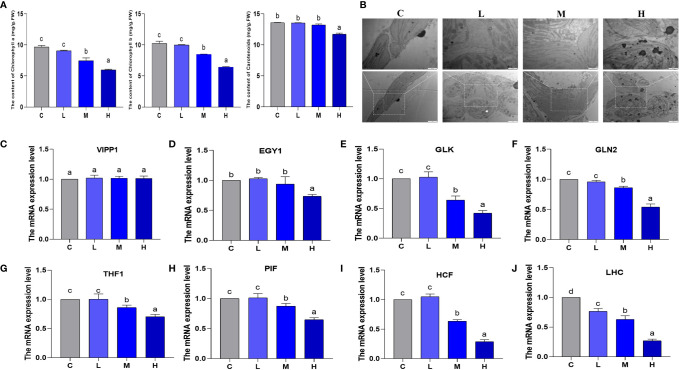
The effect of Pb stress on the chlorophyll content and chloroplast formation of pakchoi. **(A)** The content of chlorophyll a, chlorophyll b, and carotenoids in pakchoi leaves (45 d) (n=3). **(B)** Observation on the ultrastructure of chloroplasts in pakchoi leaves (n=3). Scale: 20 × (1 µm), 40 × (500 nm). **(C)** qRT-PCR analysis of VIPP1 mRNA level in pakchoi leaves (45 d) (n=3). **(D)** qRT-PCR analysis of EGY1 mRNA level in pakchoi leaves (45 d) (n=3). **(E)** qRT-PCR analysis of GLK mRNA level in pakchoi leaves (45 d) (n=3). **(F)** qRT-PCR analysis of GLN2 mRNA level in pakchoi leaves (45 d) (n=3). **(G)** qRT-PCR analysis of THF1 mRNA level in pakchoi leaves (45 d) (n=3). **(H)** qRT-PCR analysis of PIF mRNA level in pakchoi leaves (45 d) (n=3). **(I)** qRT-PCR analysis of HCF mRNA level in pakchoi leaves (45 d) (n=3). **(J)** qRT-PCR analysis of GLDH mRNA level in pakchoi leaves (45 d) (n=3). The same letter indicates no significant difference (P > 0.05); completely different letter indicates significant difference (P < 0.05).

From the perspective of chlorophyll content and chloroplast formation, we explored the effect of Pb stress on pakchoi chloroplasts. No significant changes were observed in chlorophyll a and chlorophyll b between the control and the L groups (**Figure 6A**). In contrast, in the M group, we observed that chlorophyll a and chlorophyll b content decreased by 22.7% and 38.1%, respectively; additionally, the H group decreased by 17.4% and 37.4%, respectively (P < 0.05). Hereafter, the carotenoid content changed slightly, and only slightly decreased in the H group. In the control group, the chloroplast envelope was clear and complete, and was close to the cell membrane. The substrate layer was densely arranged and clearly structured, with a small amount of starvation particles on the surface (**Figure 6B**). In the L group, the chloroplasts were swollen, and the grana lamella structure was clear and relatively complete. In the M group, the chloroplast membrane began to dissolve, and the gap between the stroma lamellae increased, showing irregular arrangement. In the H group, the chloroplast membrane was severely dissolved, the grana lamella was partially disintegrated, and the chloroplast was separated from the cell wall, resulting in more starvation granules.

Additionally, the vesicle-inducing protein plastid 1 (VIPP1) is located in the thylakoid, and the transcription level of VIPP1 did not change significantly (**Figure 6C**). Subsequently, we analyzed the transcription levels of the chloroplast development-related genes Golden 2-like (GLK), Glutamine synthetase 2 (GLN2), and ethylene-dependent geotropism yellow-green 1 (EGY1). EGY1 was significantly reduced in the high-dosage Pb treatment (P < 0.05) (**Figure 6D**). Compared with the control and L groups, the transcription levels of GLK and GLN2 in the M and H groups decreased, which appeared to be dosage dependent (**Figures 6E, F**). THF1 is a protein encoded by a nuclear gene located in the chloroplast. We found that the medium- and high-dosage Pb treatments reduced the mRNA levels of THF1 (P < 0.05) (**Figure 6G**). Additionally, phytochrome interacting factor (PIF) and high chlorophyll fluorescence (HCF) are both involved in chloroplast development and biosynthesis. The medium- and high-dosage Pb treatments reduced the transcription levels of PIF and HCF in a dosage-dependent manner (P < 0.05) (**Figures 6H, I**). The mRNA expression of the light-harvesting chlorophyll a/b protein complex (LHC) decreased, which appeared to be dosage-dependent (**Figure 6J**). These results show that the influence of Pb stress on pakchoi chlorophyll content and formation is dosage-dependent. That is, chlorophyll content and chloroplast formation decreased as the Pb dosage increased.

## Discussion

Pb residues are found in both industrial and residential soils, and are transferred to, and accumulated in plants (Doris et al., 2021; Gao et al., 2021). Pb is known to cause chlorosis, oxidative stress, and growth and development disorders in plant leaves (Kanwal et al., 2020). This study evaluated the effects of different dosages (300 mg/kg, 600 mg/kg, and 900 mg/kg) of Pb stress on pakchoi leaf chlorosis, oxidative stress, and growth and development. Pb stress caused dosage-dependent oxidative stress (**Figure 3**), abnormal mineral content (**Figures 1**, **2**), inhibition of the AsA-GSH system and photosynthesis (**Figures 3**, **4**), abnormal chlorophyll content, and abnormal expression of chloroplast development genes (**Figure 6**).

Heavy metal stress can cause a mineral imbalance in humans, animals, and plants (Lei et al., 2021; Xu et al., 2021; Bushra et al., 2022). In addition, Cd and As are known toxic mineral elements that easily accumulate in plants, and affect growth and development (Dai et al., 2019; Irshad et al., 2021). The ICP-MS method can determine the content of all elements in plant and animal tissues, and principal component analysis and correlation analysis can simplify the complex relationships between these elements (Xu et al., 2021) to reveal small-scale changes in elemental content in pakchoi leaves that experience Pb stress. This experiment found that, as the dosage of soil Pb increased, the content of the important growth elements B and Se decreased, while the content of toxic mineral elements such as Cd, As, and Cu increased. These results indicate that Pb stress decreases the absorption of beneficial elements in pakchoi leaves in a dosage-dependent manner, while the deposition of other toxic metal elements increases. Boron is a vital element for plant reproduction and growth, and plays an important role in the physiological processes of crop plant leaf expansion and meristem development (Pinho et al., 2015). The PCA showed that Se, B, Hg, Tl, and Ba belonged to the first component. In contrast, Li, K, Na, Mo, V, Co, Al, Mn, Cu, Zn, and Fe belonged to the second component. Se and B were positively correlated with component one, while Pb was negatively correlated with component one. Therefore, Se and B are negatively correlated with Pb, which agrees with the results obtained by correlation analysis. This also suggests that adding B or Se to Pb-stressed pakchoi may be used as an antagonist for Pb stress. Pb treatment can reduce Se content, while Se supplementation can also reduce Pb content. There was a negative correlation between Pb and Se (Huang et al., 2021).

The AsA-GSH system is composed of AsA (that is, vitamin C)-DHA and GSH-GSSG processes, as well as enzymes involved in these two processes, and resists environmental stresses such as low light levels (Hu et al., 2019). According to reports, Cd and Cu can accumulate in plants, thereby causing the activities of MDHAR, APX and DHAR to decrease, abnormal levels of AsA and DHA, and a decreased GSH and GSSG content, leading to oxidative stress and imbalance of the AsA-GSH cycle (Zhou et al., 2018; Jung et al., 2021). Salt-alkali mixed stress reduces the key enzymes of the AsA synthesis pathway, as well as L-galactose dehydrogenase (GDH) and L-galactose-1,4-lactone dehydrogenase (GLDH) activities, and weakens AsA-GSH cycle efficiency, thereby causing oxidative damage to naked oats (Liu et al., 2021). Additionally, ammonia gas stress decreases the activities of antioxidant systems (SOD, T-AOC, and GSH-Px), but increases the MDA concentration in chickens (Han et al., 2020). Boron (B) and chromium (Cr) stress increases MDA and causes oxidative stress in wheat (Ashraf et al., 2022). The results of this study are similar to the above-mentioned literature. Furthermore, under medium and high Pb dosages, AsA and vitamin C synthesis key enzyme (GLDH) content continued to decrease, indicating that Pb has a dosage-dependent inhibitory effect on the vitamin C synthesis of pakchoi. With an increased Pb dosage, the oxidative stress marker MDA continued to increase, and the activities of antioxidant enzymes continued to decrease. This shows that dosage dependent Pb stress causes decreased antioxidant capacity and increases oxidative stress levels. We also found that the tolerance of pakchoi to adverse environmental conditions is reduced through the GSH/GSSG ratio. Using APX and DHAR activity abnormalities, we can summarize the above results as: Pb stress causes a dosage-dependent AsA-GSH circulatory system imbalance, which in turn reduces the tolerance of pakchoi to oxidative stress.

Photosynthesis occurs in chloroplasts. The chlorophyll within the chloroplast absorbs light energy and participates in normal photosynthetic processes. The net photosynthetic rate is thus a key indicator for evaluating photosynthetic efficiency in plants. Under high-dosage metal accumulation stress (60 mg/kg Cd + 90 mg/kg Cu), the photosynthetic characteristics (chlorophyll a and b content, as well as Pn, Tr, Gr, and Ci) and nutrients of pea plants are reduced (Lei et al., 2021). This experiment found that medium and high Pb dosages reduced the values of Pn, Tr, and Gs, while high Pb dosages significantly reduced Ci values, which explained the negative effects of Pb stress on pakchoi photosynthesis. Additionally, PSII photoreaction is an important stage of photoreaction (Ci et al., 2009), and Fv/Fm and Fv/Fo values can be used to measure the original light energy conversion efficiency and maximum light energy conversion potential of the PSII system in pakchoi. Under 100 μmol/L Cd hydroponic conditions, the Fv/Fo and Fv/Fm photosynthetic parameters of elsholtzia serrata are significantly reduced (Li et al., 2015). This study found that Fv/Fm continued to decrease as Pb dosage increased, which thus showed a dosage-dependent effect. Pb stress thus negatively affects pakchoi photosynthesis *via* the abnormality of PSII light response system. Additionally, the effects of the medium and high Pb dosages on the contents of chlorophyll a and chlorophyll b were reduced in a dosage-dependent manner. The ultrastructural observation of chloroplast showed that with the increase of Pb dosage, the integrity of chloroplast and stromal sheet was destroyed, which would directly affect the photosynthesis and chlorophyll content. GLK expression is known to lead to increased levels of chlorophyll and LHC (Li et al., 2020), and genes such as PIF and HCF are also involved in chloroplast development and chlorophyll synthesis (Schmitz et al., 2012; Zhang et al., 2021). The EGY1 (Ethylene-dependent gravitropism-deficient and yellow-green 1) gene encodes for a thylakoid membrane-localized protease involved in chloroplast development in the mesophyll cells (Sanjaya et al., 2021). Our research also found that after Pb stress, the expression of pakchoi chlorophyll synthesis (HCF and PIF) and chloroplast development-related (GLK, GLN2, and EGY1) genes were down-regulated to varying degrees, thereby further confirming that Pb stress may affect pakchoi photosynthesis through chloroplast development and the downregulation of chlorophyll synthesis. These results indicate that Pb exposure affects the PSII photoresponse system by affecting chloroplast development and chlorophyll synthesis in a dosage-dependent manner.

In conclusion, we found that Pb stress has an adverse dosage-dependent effect on the mineral content of pakchoi, as well as AsA-GSH and photosynthesis. Thus, Pb induces oxidative stress in pakchoi in which photosynthesis and the AsA-GSH cycle are weakened, which further leads to abnormal chlorophyll content and decreasing chloroplast development gene expression. Heavy metals accumulate in plants through the environmental food chain, and thus threaten human and animal health, and eventually the entire ecological environment. The results of this study supplement the toxicology of heavy metals, and provide instructions for pakchoi cultivation and related warnings for heavy metal hazards.

## Data availability statement

The datasets presented in this study can be found in online repositories. The names of the repository/repositories and accession number(s) can be found in the article/**Supplementary Material**.

## Author contributions

ZT: visualization, investigation, writing-original draft. CW: manuscript revision and formal analysis. ZX: software and formal analysis. YC: formal analysis. RX: software and investigation. ZS: software and investigation. DW: conceptualization, resources, supervision, validation, and writing-review and editing. All authors contributed to the article and approved the submitted version.

## Funding

This work was supported by Xinjiang Production and Construction Corps Programs for Science and Technology Development (2018DB003) and Innovation and Entrepreneurship Platform and Base Construction Project of XPCC (2019CB001).

## Conflict of interest

The authors declare that the research was conducted in the absence of any commercial or financial relationships that could be construed as a potential conflict of interest.

## Publisher’s note

All claims expressed in this article are solely those of the authors and do not necessarily represent those of their affiliated organizations, or those of the publisher, the editors and the reviewers. Any product that may be evaluated in this article, or claim that may be made by its manufacturer, is not guaranteed or endorsed by the publisher.
