# Supplementary material for: Lead exposure dose-dependently affects oxidative stress, AsA-GSH, photosynthesis, and mineral content in pakchoi (Brassica chinensis L.)
Source: Front Plant Sci. 2022 Oct 6;13:1007276. doi: 10.3389/fpls.2022.1007276 (PMC9583015; doi:10.3389/fpls.2022.1007276)
Supplement: Supplementary file 1 [file Table_1.docx]

**Table S1 Gene primers used in qRT-PCR**

| Gene | Forward Primer (5’-3’) | Reverse Primer (5’-3’) | Gene ID |
| --- | --- | --- | --- |
| VIPP1 | TGTTGCAGCAAGCACGAGATACC | AGTCGTTAGCTTTCCTTCGCAGTTC | 106322547 |
| EGY1 | CTCTTATCCACCATTTCCGCCTCAC | CCAGCTTCCCTCCACGAATTGC | 106300889 |
| GLK | GACGGTGACTTATGACGGTGACAG | ATCTCCTGTGTAGCTCTGGTGTCC | 834442 |
| GLN2 | GTGACTGGAACGGTGCTGGTTG | GGCGAAGCGAGAGGTTCAAGATAG | 833535 |
| THF1 | TTCTTCGCTGTTGGGCTCTTTCG | GCAGGTTACGATACACATCCAGGTC | 816623 |
| PIF | TTCCGCATCCACATCTTCACAGTC | TGTTGACGGCGAGGAAGGAGAG | 818903 |
| HCF | ATCAATGGCGTCTCTGCAACTCTG | GGAGATGGAGGAGGAGAAGATGAGG | 838243 |
| LHC | CAACCCAAACCTAGTCCATGCTCAG | TGCCTTCGCCAACACCATCAAG | 815055 |
| IRT1 | CACGAGCCTATACACCAGCAAGAA C | ATGGCAATGACTCGGTATCGCAAG | 827713 |
| IRT2 | TTCCTCACTGTCGTCTACTCCTTCG | CTCCTTGGCTAGTGGCATTGATTCC | 827712 |
| APX | ACCAAGGGTTGTGACCATTTGAGAG | CAGAGTGTGGGCACCAGATAAAGC | 106294764 |
| DHAR | TGAGAGCTTGACCAGTGTTCGTAAC | TTCCCAACCCGCAACCACAATC | 106315491 |
| GLDH | AATGGGATTGGTTTGTCTCGCTCTG | GCCTGCACCGTAACTCTCTTCTTC | 823948 |
| β-actin | GACTACGAGCAGGAGATGGAGACC | ACGAACGATGGCTGGAACAAGAC | 106295461 |
