# Supplementary figures and images for: Lead exposure dose-dependently affects oxidative stress, AsA-GSH, photosynthesis, and mineral content in pakchoi (Brassica chinensis L.)
Source: Front Plant Sci. 2022 Oct 6;13:1007276. doi: 10.3389/fpls.2022.1007276 (PMC9583015; doi:10.3389/fpls.2022.1007276)

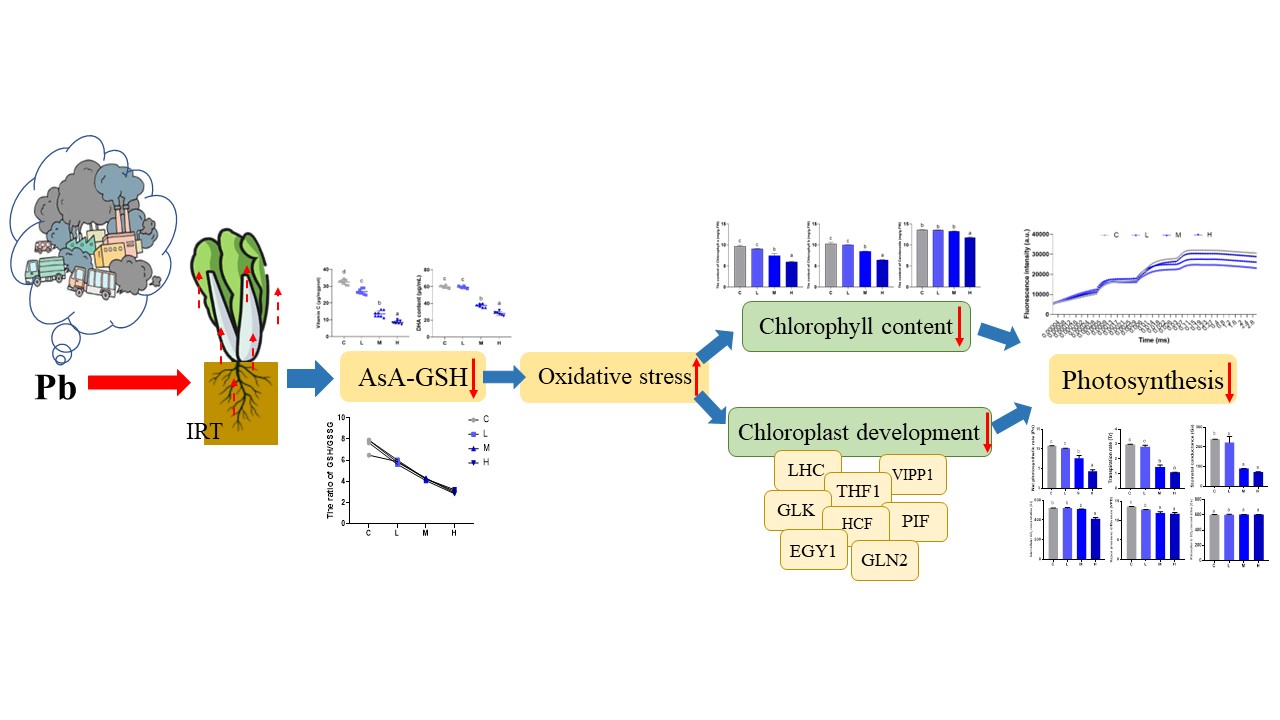

Supplement: Supplementary file 3 [file Image_1.jpeg]
